# Synthetic Life and the Value of Life

**DOI:** 10.3389/fbioe.2021.701942

**Published:** 2021-06-17

**Authors:** Erik Persson

**Affiliations:** Department of Philosophy, Lund University, Lund, Sweden

**Keywords:** value, end value, synthetic life, originality, origin of life, mystery of life, naturalness, autonomy

## Abstract

If humans eventually attain the ability to create new life forms, how will it affect the value of life? This is one of several questions that can be sources of concern when discussing synthetic life, but is the concern justified? In an attempt to answer this question, I have analyzed some possible reasons why an ability to create synthetic life would threaten the value of life in general (that is, not just of the synthetic creations), to see if they really give us reason to worry. The main conclusion is that it is unlikely that a future human ability to create life will really have a great negative impact on these characteristics of life. It therefore seems unlikely that the value of life will be negatively affected by the ability to create synthetic life, though it is possible that the properties in question will be less salient in the synthetic life and thus that the value of the synthetic life will be lower than that of existing life, which in turn can lead to a disturbing difference in value between different kinds of life.

## Introduction

Will the value of life be negatively affected if human beings acquire the ability to create life from non-living material? In this article, I will take a close look at some phenomena that have three things in common: They are closely associated with life, they are commonly seen as conferring value to the entities that possess them, and they may be negatively affected by a human ability to create life. The purpose of this investigation is to find out whether each of these properties of life are threatened by a future human ability to create life, and if that will threaten the value of life. The study concludes that even though some of these properties may be questioned in the new life forms, this will not affect the value of existing life or of life in general.

The characteristics I will take a closer look at are the originality of life, the origin of life, the mystery that surrounds life and its origin, the seemingly obvious fact that life is a natural phenomenon, and the ability of life to create itself and to find its own way, that is, its autonomy. In addition, I will briefly discuss the diversity of life as an example of a trait that is highly valued, and that may actually be strengthened by a human ability to create life if it leads to the creation of more, and more different, life forms.

Before we start the investigation, let me explain a little more about what I mean, and what I do not mean, when I talk about the value of life. What does it mean that life has value? As I imagine it, it is about our attitudes toward life. The vast majority of people seem to have a generally positive attitude toward life. Of course, this does not prevent one from having a negative or neutral attitude toward particular individual lives or life forms. One may be of the opinion that a certain life, or even a certain type of life, a life in constant pain and without hope of relief, for example, is a negative life that one would rather end. Most of us are probably also of the opinion that it is perfectly acceptable to end the lives of millions of bacteria with the help of antibiotics, or for that matter, every time we wash our hands, to protect our own lives. None of these cases, however, prevents us from thinking that life is, basically, something good. It can manifest itself in everything from being happy that life arose to devoting one’s own life to protecting the lives of others or aspiring to spread life across the galaxy. If life is something good, why not make sure it exists in as many places as possible in the universe? Maybe it also means a curiosity about how life originated, how it works and if there is life outside our planet. Some may even see the positive value of life as an argument to create more types of life, that is, synthetic life. In a more philosophical language, one might say that life has value if you, all other things being equal, think that life is something good. How that value stands in relation to other values depends on how valuable one thinks life is and how high the competing values are, but I will not go into that here. I will also not talk about the life of a particular individual and I will not limit myself to any particular kind of life, such as human or mammalian life. The value I am thinking of here is something more fundamental. It is not about the value of specific living individuals, but about the value of life itself. In other words, a value that is independent of which living being we are talking about. When I talk about the value of life, I am not talking about the value of you, your cat, or your potted plants, but about the shared fact that you are all alive.

Value is a concept that comes in many different forms. When I talk about the value of life, I only refer to so-called, end value, that is, whether life has value as an end in itself (Independently of whether it also has value as a means to something else) to a valuing entity, for instance, a human being. I am not talking about instrumental value, economic value, or indeed about moral status.

Economic “value” (price) is a very different concept than the kind of value I am talking about here. We can illustrate the difference by comparing the value and the price of a piece of art. The most valuable works of art are considered economically “invaluable,” not because they could not be sold (some collectors are willing to pay very large sums of money for works of art that they will never dare to show or admit that they have) but because their value cannot be properly stated in monetary terms. If, for example, someone wanted to buy a unique work of art, say, Mona Lisa, with the aim of destroying it, I suspect that it would lead to extremely upset feelings no matter how much money the buyer is willing to pay for the “fun” of destroying it.

Moral status is also, but obviously for different reasons, something completely different from value – of any kind, including end value. Unfortunately, the question of someone’s or something’s moral status is all too often confused with or conflated with the question of its value. As I prepared this article, I found this to be the case also in connection with discussions about the value of life and about synthetic biology, which made it more difficult that it should have been to make sense of some of the literature in the area. I find it important, however, to keep the two apart and to stress that someone’s moral status is completely independent of that someone’s value. Moral status is something you have by virtue of having interests that others need to take into account when they act. That is, you have moral status if and only if things can have (positive or negative) value to you. In a more technical language, you need to be a subject who can experience things in a subjective way as being positive or negative. If a being does not have subjective experiences that can be classified as positive or negative for that being in at least a rudimentary sense, then it is very difficult to understand what it would even mean that this being has interests and even more difficult to see what it would mean to respect these interests.

Value is different. An antique vase can have a very high value, regardless of whether it experiences something itself. If the vase falls to the floor and breaks, it means a loss of value even though the vase does not feel any pain and does not feel offended or sad that it will no longer get to experience the admiring glances of future guests. The vase, therefore, has no moral status but it has value. If we have a moral duty not to crush the vase or even to protect the vase from being crushed by others, it is because it has value to other valuing entities, perhaps to its owner or perhaps to everyone who visits the museum where it is exhibited, and maybe to humans who have never seen and will never see the vase if the vase, for example, is part of our human cultural heritage.

This moral imperative to protect what has value to other moral objects also means that even though end value and moral status are not the same thing, and even though end value does not imply moral status, doing something that would diminish the end value of life is still a question of moral significance.

When talking of the value of life in this text I do not intend to estimate how valuable it is, either in absolute terms or relative to other things. I leave this exercise entirely up to the reader. Maybe you are of the opinion that life itself has no value at all. Maybe you are of the opinion that life is something that is extremely valuable and must be protected and preserved at all costs, or, perhaps most likely, you are somewhere in between. No matter where you are on that scale, however, you can always ask the question of whether the value will be reduced on the day we humans learn to make life on our own. This is true even if you think that life has zero value. After all, no value at all is better than a negative value.

One difficulty when talking about the value of life is that there is no generally accepted definition of life. It is, of course, a challenge in itself to talk about the value of something that one does not fully agree on what it is (see, e.g., Abbott and Persson, 2021). I will not go into this question in more detail here as it would lead too far from the main question. One thing that needs some elaboration, however, is the meaning of the phrase “creating life,” which is central in this article. When I talk about creation of life or created life, I refer to the deliberate process of making new life from non-living material according to principles of engineering. I do not refer to the propagation of life that goes on all the time and where all the new lives directly descend from already existing life. I am also not talking about modification of existing life, as is already done in different ways ranging from surgery via genetic modification to the substitution of an entire genome that was done at the J Craig Venter Institute in 2010 (Gibson et al., 2010). Instead, I am talking about a new origin of life, where life, not related to existing life is engineered from entirely non-living material.

## The Originality of Life

The way we value life is sometimes compared with how we value art (e.g., Elliot, 1982). Can that help us in this situation? Originality is commonly seen as a reason to attach high value to, for example, a work of art. We tend to value works of art higher if they are original (e.g., Kozbelt, 2004). If something is significantly different from everything else, it also becomes more interesting. A work of art that differs radically from all other works of art, or even from all other objects in the world, creates interest and increases the value of the work of art, often regardless of whether it is considered beautiful or not. Works of art that are less original are considered less valuable than art that is more original (other aspects being equal). Things that are mass-produced have, from this perspective, a much lower value even when they are as beautiful as an original painting or sculpture.

How does this relate to the question of the value of life? Does it mean that if we learn to create life and thus get multiple editions of life, it will diminish life’s value because life will no longer be a unique phenomenon?

What we have to ask ourselves here is whether life is really original in the same way as a work of art, whether it is really from there that life get its special value, and if this originality will be diminished if we start producing life with a different origin than existing life.

All living beings differ from all other living beings in some way, but this applies to everything and can hardly be a reason to assign any extra high value to it (What would “extra high value” even mean if everything has it?). Besides, many living beings are also similar in some respects. That is why we can divide life into species, genera and so on. It is even the case that all life on earth today is related, albeit distantly so. We all descend from a common ancestor, the Last Universal Common Ancestor (LUCA). Admittedly, this means that each of us is not as original as we might like to think, though, relative to things that are not alive, one might still claim that we are quite original just by being alive. Exactly how much life as we know it today differs from non-life is a question that largely depends on how we define “life.” Defining “life” is not an easy task, however, (e.g., Persson, 2013; Abbott and Persson, 2021), which makes it difficult to base the value of life on how and how much we differ from the non-living. The fact that all life on earth originates from the same “ancestor” means on the other hand that we living beings together differ clearly from everything that is non-living, namely through our common origin. Can this fact be used to assert the originality of life? If life has only arisen once, then we can rightly say that life is really original.

However, the answer to the question of whether life has only arisen once is that we do not know. It may be that life on Earth has only arisen once, but it may also be that it has arisen several times but that only one of the times has succeeded in the sense that life managed to survive and spread across the planet. In addition, we do not yet know if there is life outside our planet.

If life has only arisen once in the history of the universe, then we can really say that the life we know on Earth is original, even unique, in the same way as a unique work of art, like the Mona Lisa for example. If instead, it is the case that life has arisen several times, then it still seems to be the case that “our” life is unique in the sense that it is the only now existing life on Earth. It means it is not unique in the universal sense that it is the only life in the universe, but it will be the only life on Earth and probably the only life of its kind in the universe, so it is still original enough to be assigned a very high value. We might say that it has value in the same sense, and to the same degree, as an archeological finding of an object of which there were once several specimens but of which there is now only one left, and of which there may be several preserved specimens in other countries, but where the exact specimen we have in front of us is the only preserved specimen in our country. Even in that case, however, it seems justified to attach a rather high value to it based on its originality, even though it is then not entirely unique from a broader (geographical and temporal) perspective.

One can also reason that the physical and chemical conditions on different planets are quite different, so if there is life on other planets, that life is probably quite different compared to life on our planet. If we compare with works of art again, there are a lot of invaluable works of art on Earth today, but each of them is original relative to the others. They are all works of art, but they are sufficiently different from each other to be given a special value. Maybe we can say the same about life on different celestial bodies? If the universe is full of life, but life on each planet is different enough from life on any other planet, we might still be able to say that each type of life is original enough to deserve an extremely high value.

Our next question is, does the value of life depend on its originality? This question is not entirely easy to answer. When I write this, we only know of one instance of life and have never experienced a situation where there have been several editions of life (that is, life with different origins). What we can say is that even though every life form we know of today has a common origin and even though we all have a lot in common, there is also a large degree of variation. Maybe this variation within the category of life, with each life form showing a high degree of originality within the class of life, is one of the aspects of life that makes us value life as such so much? This interpretation of the role of originality in the value of life is a bit of a stretch from the original concept (originality among life rather than originality of life) but it seems to make sense. The diversity of life is often mentioned as an important reason for why we value it so highly (Kleinig, 1991). We could even twitch our comparison with art, and instead of comparing life with Mona Lisa, we can compare life with art as such, in which case, maybe the highly valued originality among works of art is one of the reasons we tend to value art so much?

Is it reasonable to expect that the originality of life (in either of the senses above) will diminish if we learn to artificially create life? Let us go back to the comparison of life with a work of art. We previously stated that a mass-produced work does not have the same value as a work of which there is only one copy, for example, the Mona Lisa. On the other hand, there are actually a very large number of reproductions of the Mona Lisa. You can even buy postcards with the Mona Lisa motive in the museum shop at the Louvre. So, there is actually a myriad of Mona Lisa copies. What does this mean for the value of the Mona Lisa? In order to give a sensible answer to this question, we must ask ourselves what it is we are referring to. Is it Leonardo’s original painting, is it the copies, or is it both? If we start with the copies, they do not seem to be valued very highly. They can be bought quite cheaply, and we would not be terribly upset if a postcard with a Mona Lisa motif disappears. So, what does the existence of all these Mona Lisa copies mean for the value of the original painting? The answer seems to be: Nothing. No matter how many copies we make of the Mona Lisa, this does not seem to in any way diminish the originality of the original and thus not its value. It is part of the very concept of “originality” that the original is always the original no matter how many copies are made. The fact that the copy is a copy makes its value smaller, but it does not diminish the value of the original.

How does this reasoning transfer to the value of life? It seems to imply that if we succeed in making several instances of life on earth with us as creators, these copies will have a lower value, at least from this special perspective, while the original is not affected at all. It is still the original and it retains its value.

If we instead compare life with art as such and derive value from the originality of the multitude of different life forms instead of the originality of life itself, the situation may be different. An addition of life forms in the form of synthetic life would not make life less multifaceted. We might even be justified to claim that it would add originality, both by adding more original life forms, and by adding life forms that are more original (by having a different origin). On the other hand, we can safely say that the created life will initially contain much lower diversity than the original life, which would reinforce the image of a class difference between the two. In any case, this should be easy to fix by allowing, or even encouraging, the creation of many different artificial life forms. Whether this is advisable from other perspectives is of course another question.

Whether it really works to make such a comparison between life and art is difficult to answer, which is probably due partly to the difficulty of identifying what it is that is so special about life to begin with. In part, it is probably also because we do not have any synthetic life around today and therefore cannot know for sure how our values will change once such life appears, but it would of course be good to have a hint of an answer before we start making copies. Once they exist, it will be too late to change. The reasoning about originality and the comparison with art does not seem to give a strong indication that the value of existing life would decrease if we learn how to copy it, but perhaps it can be a problem if it leads to the new, artificial life being assigned a lower value than the original. On the other hand, we do not know how crucial this lack of originality will be.

The question of whether the new life can be said to add internal originality to life and thus increase life’s value is not entirely easy to answer.

## The Origin of Life

Another reason why we value some works of art more than others is because of their history and the work and skill it took to create them (e.g., Elliot, 1982). This is something that is not only true for art. We generally tend to value things higher if they are difficult to achieve and we tend to value things higher if fewer people can accomplish them. Is the same true for life? So far, no human has succeeded in creating life, and that is not because we have not tried. If one of the reasons why we value life so highly is that it is very difficult to make from scratch, then how will the value of life be affected when we learn to create it?

The life that exists on earth today, “life 1.0,” differs, of course, from works of art in that it has not been created in the same way as works of art, and above all in that there is no artist behind the creation (Elliot, 1982) (I assume here that there is no divine creator behind the emergence of life. If one believes that life as we know it has a creator, then there is at least no human artist behind the creation of life).

Can the origin of life then be compared with the creation of a work of art even if there is no creator of life as we know it today? It is difficult to talk about things like skill and creativity when there is no creator, but our task is not to identify any skilled or creative artist behind life 1.0 that we can honor. Our task is to find out what gives life its value and whether it is diminished if humans seize the ability to create new life. If we return to the Mona Lisa, it can be said that we have reason to admire both the work and the artist. When it comes to life 1.0, we have no artist, but can the way in which it has originated still give us reason to admire “the work,” that is, life?

We do not yet know exactly how life came into being, so how can we say that the circumstances of its origin give us reason to attach special value to it? One answer to that question may be that the very fact that we have not yet figured out how it happened indicates that it was not a simple process. For us to achieve the ability to create life is thus something that must require an enormous amount of work and probably a real dose of genius. Nobody has done that so far. This means that the analogy with works of art can still hold. Even though the work of art differs from life 1.0 in that the former has an artist while the latter has none, life still has in common with the most valuable works of art that they are things that not everyone can create. If at least part of the value of life is based on it being so difficult to create that no human has managed to do it, then it looks like we have to accept that the value of life will diminish if we learn how to create life from scratch.

As in the previous section, it is important to distinguish between how a human ability to create life will affect the value of the created life and how it will affect the value of existing life and life in general. We noted in the previous section that no matter how many copies there are of a work of art, the original is still the original. In the same way, no matter how many people copy something, it is always a greater achievement to be the first to do something. Kleinig (1991) argues that it is not originality *per se* but the “manifestation of creativity” that gives original works of art a value that copies lack. If someone succeeds in copying a work of art, it is still just a matter of copying. Making a good copy can be very difficult and require a skill that few can fully acquire, but it cannot be compared to the skill and creativity required to be the first to come up with something. The same goes for inventions and scientific discoveries. The inventor or discoverer is honored much more than those who make copies of the invention or repeat the experiment or observations behind the discovery, and rightly so. Doing something for the first time, from the beginning, without someone to copy or learn from is an extraordinary achievement. This indicates that the creation of artificial life will not affect the value of the current life, but that it could affect the value of the synthetic life negatively.

On the other hand, it can also be argued that given how difficult it is to copy life, copying it must also be regarded as an incredible achievement that requires a lot of knowledge and great skill. If a thing that can never be copied is worthy of great respect, a thing that takes more than 4 billion years to copy deserves almost as much respect. This in turn indicates that even if the value of the synthetic life is lower than the value of the original, it is still very high. The researcher or research group behind the first instance of synthetic life will have made a fantastic scientific achievement. It will, of course, be an achievement based on an intensive study of life that already exists. In comparison with existing life, it will therefore remain a copy, but in comparison with other human achievements, the first creation of artificial life will still be one of the more impressive achievements in the history of our species. If the history behind the origin is important for something’s value, this also means that even if synthetic life will not have the same value as existing life, it will have a higher value than almost all other human creations.

Maybe the knowledge of how to create life will eventually become publicly available and maybe after a few years it will not require much skill or any creativity to create life anymore, but even when that is the case, life will still be one of the natural phenomena that it has taken humans the longest time to copy, which should make it only marginally less valuable compared to something that has never been copied, and much more valuable than anything else that has been copied, including all the artworks of the world.

## The Mystery of Life

It has sometimes been argued that one of the things that makes life special is that we do not really understand it. Even though knowing how to make life is not the same as understanding life, or even how it actually originated on Earth, it is probably safe to say that when we talk about such a biologically and technically complex challenge as creating life, it implies a rather deep knowledge about life. This means that if we manage to learn how to create life from scratch, that would for sure take away some of the mystique. In that case, the very knowledge required to create artificial life would cause a loss of value, even if it is not actually used to create life. The knowledge as such would take away some of the mystery and thus some of the value of life.

Similar to what we have seen before, there would be a difference between life 1.0 and life 2.0. That is, between the life that exists on earth today and the synthetic life. The difference, in this case, is that even if we succeed in figuring out how to create life, it does not necessarily mean that we have answered the question of how life actually arose on earth. Strictly speaking, it would only mean that we have found one way in which life can arise. We still cannot know for sure if the life that now exists on Earth originated in the same way. It is conceivable that there is more than one way in which life can arise. It is most likely also the case that what is required to create life in a laboratory on Earth today differs at least in some ways from what was required for life to arise spontaneously about 4 billion years ago when the Earth and its atmosphere looked completely different from today. The ability to create life would undoubtedly help us dispel some of the mystery surrounding how life 1.0 originated, but it would not remove it completely in the same way that would be the case for life 2.0, whose origin would be described in the smallest detail by its creator. This means that if mystery is important for the value of life, then we are, again, in the situation that we need to distinguish between the value of original life and the value of synthetic life. For the synthetic life, there would be no mystery surrounding its origin, while the mystery surrounding the original life and its origin would only be dispelled to some extent. The value of life 1.0 would therefore be affected, but to a lesser extent than the value of the synthetic life that arises thanks to our knowledge of how to create it.

Is the mystery surrounding the origin of life important for its value? Life can, just like art, be surrounded by mystery in many different ways (What is really behind Mona Lisa’s difficult-to-interpret smile and what were the circumstances around its theft?). The mystery surrounding the origin of life is only a part of the mysteries of life. It is certainly not a small part, but is it the only mystery that determines its value? In the case of the Mona Lisa, that does not seem to be the case.

Perhaps it is more appropriate to look at how mystery is connected to value in the case of magic than in the case of a painting? Magic is a form of performing art where mystery around the execution is the very core of its value. On the other hand, if we witness a fantastic and seemingly incomprehensible magic trick and we, after much thought, manage to figure out how it was done, what does that mean for our fascination? I cannot help but suspect that it would actually lead to us remembering that night at the theater with even greater satisfaction than if we never manage to figure out how the trick was carried out. However, we would certainly be disappointed if someone told us about it and thus deprived us of the chance to figure it out ourselves. In that case, however, it does not seem that we are deprived of the mystery, but of the chance to challenge our ability to solve it.

There are also many cases where it is obvious that more knowledge makes us value something higher. People who work with nature conservation often point out (personal communication, see also Elliot, 1982) that one of the best ways to persuade people to take better care of a nature area is to teach them about the species, ecology, geology, and not least, the history of the area.

The role of mystique as a value creator is thus not entirely unambiguous. Even though it is certainly true that mystery can make us value something higher, increased knowledge, especially knowledge that explains something we have thought about hard and long, can also make us value it higher. Also, it seems to differ from person to person. My personal experience is that the more I understand a phenomenon, the more exciting it becomes. This is, of course, a personal reflection and it is quite plausible that for others, “de-mystification” is actually a problem. The fact that this varies from person to person will, on the other hand, also make it less of a problem. If someone feels that the understanding required to create life will diminish the value of life for them, then they will probably not be willing to invest the time and effort that will undoubtedly be required to achieve this type of knowledge about life. Perhaps they will even actively avoid exposing themselves to such knowledge. This means that as long as it is voluntary to learn about life to the degree required to recreate it (which would reasonably far exceed what, for example, may be required to graduate high school even with top grades), and as long as it is all about the personal feeling of mystery, it will be easy to opt-out of this knowledge for those who so desire and continue to marvel at the mysteries of life.

It is a different issue if one’s valuing of life is diminished by the mere knowledge that someone knows enough about life to recreate it. This is undoubtedly a possible position, but it seems quite extreme, and it would be even more extreme to demand that others stop researching a topic for that reason. To stop investigating something because there are those for whom it is perceived as negative that someone (even if it is not themselves) know how it works, would be going way too far. This is especially true considering that many others (not just me) will actually be influenced in the opposite direction and see life as even more exciting and amazing thanks to this type of knowledge.

## The Naturalness of Life

Another possible reason why the creation of synthetic life mightreduce the value of life could be that the resulting life will be unnatural in some sense (Siipi, 2008; Bedau and Triant, 2009). It is a common idea that what is natural has higher value than what is unnatural (Elliot, 1982; Angermeier, 2000; Rolston, 2001; Siipi, 2008; Ode et al., 2009; Lustig, 2013; Román et al., 2017. See Kleinig, 1991; Bedau and Triant, 2009; Lustig, 2013 for a critique of naturalness as conveyor of value). Life in its original form may be considered the archetype of naturalness. Life that has been modified and thus in some sense has become less natural, however, is already today viewed with skepticism by some. If the naturalness of life is something that contributes to its value, then perhaps the advent of truly synthetic life will diminish the value of life.

The term “unnatural,” like its opposite, “natural,” is notoriously difficult to define. It is particularly difficult to define in a way that is reasonably informative *and* normative at the same time. That is, it is difficult to define in such a way that it specifies what is unnatural and why, while at the same time explaining why being unnatural would mean being less valuable.

There are hundreds of definitions of “natural” and “unnatural,” and it is impossible to go through them all here. One criterion of unnaturalness that is included in many definitions and that would be relevant in our case is that something is man-made (Hunter, 1996; Rolston, 2001; Siipi, 2008; Vining et al., 2008). It does not have to mean that man as such is unnatural. It seems that many people’s intuitions about the boundary between natural and unnatural go precisely at what humans do, not at being human as such. In many cases, it also seems to be a matter of degree rather than “either-or.” That is, things can be more or less natural (Angermeier, 2000; Siipi, 2008). This way of reasoning implies, for instance, that a dog that has come about through breeding would be less natural than a wolf but more natural than a dog that has come about through genetic modification. Breeding means that we intervene in nature by selecting the dogs for breeding that best match our ideals. We thus take over the role of “natural selection,” which accelerates evolution and guides it in a certain direction, desirable to us. Genetic modification means that we take another step by also (at least in part) taking over the role of creating the genetic changes on which the selection is based. To create life from scratch, according to this reasoning, would be to take another long step from the natural, and probably reach as far as one can come in terms of unnaturalness when it comes to life.

However, it is far from obvious that it makes sense to make this kind of distinction. It is not clear why a higher degree of human influence on a process makes it less natural if we at the same time accept that humans are part of nature. It is also unclear how to connect naturalness and value. If naturalness is only a way of indicating how little humans have been involved, how do we go from there to saying that more natural is better than less natural? In many cases, human influence has certainly led to problems in nature, but at the same time, it is difficult to deny that many human inventions in, for example, medicine and technology have actually been very useful and made our lives better. We also must not forget the human inventions to which we attach value in itself, such as art and literature. It thus seems that there are at least some obvious exceptions to the assumption that human impact is necessarily negative. This indicates that human involvement as such is maybe not a good basis for value or disvalue. It can therefore be difficult to maintain a general principle that the more human impact, the lower the value. Using “naturalness” as a fancy term for “human involvement” does not seem to change this. It can even be perceived as a way of trying to obscure a dubious connection by using fuzzy terminology.

If we still accept that the degree of human influence determines how natural something is, and that being less natural also makes something less valuable – two assumptions that as we saw, are far from obvious – then the question remains what this means for the value of life if and when we learn to create synthetic life.

Again, we must distinguish between the synthetic life (life 2.0) and the original (life 1.0). If we accept that more human intervention means less naturalness and thus lower value, then it becomes obvious that synthetic life becomes less natural and thus less valuable than the life we know today that arose long, long before humans entered the scene. Thus, we again need to distinguish between the value of the new, synthetic life on one hand, and how the emergence of synthetic life will affect the value of existing life. The perceived connection between naturalness and value will affect the synthetic life much more than the original life, which in turn leads to a worrying difference in value between them. One might even suspect that synthetic life will serve as a contrast to the original life and make it seem even more natural, which could make the difference even greater, at least in our minds.

However, we should also look at things from a longer perspective. What happens after a few generations when the new life is not that new anymore and the organisms created by us have started to multiply on their own? Will all life that descend from synthetic organisms be unnatural forever, or will the organisms that descend from the first synthetic organisms gradually begin to be considered more natural because these later generations have arisen and evolved through the laws that govern all life, original as well as synthetic? [Douglas et al. (2013) answers this question in the negative].

In summary, it can be said that if we base value on degree of naturalness, it points in the same direction as most of the properties we have discussed above, that is, even if we seize the ability to create life, it will not significantly affect the value of existing life, but it might create a gap between the new and the old life, which can be a source of concern. However, we must also remember that naturalness seems to be a highly dubious trait on which to base the value of life.

## The Autonomy of Life

Is it possible that if we learn to create life, we take over a power that properly belongs to life itself, and that in doing that, we take away some of nature’s value? It is sometimes said that we “play God” when we manipulate life and even more so if we learn how to create it from scratch (see Bedau and Triant, 2009; Boniolo, 2009; Douglas et al., 2013; Kaebnick and Murray, 2013; Link, 2013; Melin, 2021 for discussions). The purpose of the “playing God-objection” is usually to convey either that even if we have enough practical knowledge to do something it does not imply that we also possess enough wisdom to use it in a safe way, or that we have just overstepped a boundary that should not be overstepped. It is certainly true that through synthetic biology, and in particular by acquiring the ability to create life from scratch, we will acquire “a degree of control over the basic mechanisms of life that human beings have never attained before” (Kaebnick and Murray, 2013), and it does indeed require us to ask with Kaebnick and Murray (2013): “is that quest desirable? Is it troubling?”

The use of the phrase “play God” in the sense that we lack the wisdom to fully understand the consequences of what we do is mirrored in many literary works ranging from Frankenstein (Shelley, 1818) to Jurassic Park (Crichton, 1990). In these cases, however, it seems that the problems are of a different kind than the one we discuss about the value of life. In the case of Frankenstein’s monster, it seems that the real problem is that Frankenstein do not take proper care of the life he created (Cabak Redei, 2021), and in the case of Jurassic Park, it seems that John Hammond and his bioengineers overestimate their ability to control the life they create. Both of these literary works provide good reasons to think twice before we set out to create (or, as in Jurassic Park, recreate) life, but they do not seem to have anything to do with the value of life as such.

The other sense of the phrase “play God,” that is, that we have overstepped some boundary that we are not supposed to overstep, can be interpreted either theologically or secularly. When used in a secular sense, it is common to mean that we take over a power that in some sense belongs to nature and that this overtaking can negatively affect the value of nature. Elliot (1982) exclaims in his critique of nature restoration projects: “We value the forest and river in part because they are representative of the world outside our dominion because their existence is independent of us” (Elliot, 1982).

Can this line of thought, motivate the feeling that if we acquire the ability to create life, we acquire a power that rightly belongs to life itself and that acquiring this power diminishes the power and thus the value of life?

A general difficulty with the argument that we take over the power to create life from life itself is that it is a bit unclear how it should be interpreted. Life has the ability to create more life, but it has never had the ability to create life from scratch. Life did not exist when life first arose. In fact, one could even turn the argument around and say that once humans acquire the ability to create life from scratch, this actually means that life (in the form of human beings) for the first time on our planet acquires the ability create new life (not just more life). We could thus say that through us, life acquires the power to create life. Humans learning to create new life will thus increase the power of life over itself rather than reduce it.

However, we can also look at this from an equality perspective. By learning to create life, we “give” life an ability that no life has had before, but it is an ability that will only be possessed by one life form – humans (and not even all humans). It thus widens the already existing immense power gap in nature between our species and all other species. How much difference this added power makes in practice is difficult to speculate about. The fact is that we already, to a large extent have the power to control the living conditions for virtually all life on earth. There are of course worrying practical implications of this power but that is another matter. In the worst case, perhaps the new life might become invasive and thus will pose a threat to existing life. At best, perhaps the knowledge we gain through our attempts to create synthetic life make us better at taking care of existing life, though not much indicate that this is how we are going to use that knowledge. Guiding research and development in the right direction is an extremely important challenge, but it does not seem to be about the value of life as such, but rather about how we best take care of life given that it actually has a high value.

However, there is another aspect of this that may give cause for concern. Some believe that the new life will be seen as machines or goods and that this will spread to all life, including the original life (see Bedau and Triant, 2009; Douglas et al., 2013). This is an attitude to life that can really cause concern. The idea is that by acquiring the ability to produce life at will, we will come to consider all living organisms as products among other products. This is a way of taking control of life that would be clearly negative.

One can object here that living beings are to a large extent already regarded as products and raw materials in this way. How non-human animals are regarded and treated in society as a whole, as resources, entertainment and more, is one of the greatest and most offensive moral failures of our species. The real question here, however, is whether this will worsen with the advent of artificial life. We cannot deny that there is a risk to that. On the other hand, we have also seen that society’s norms generally move slowly toward being more inclusive. More and more groups have come to be included in the moral community and this also applies (though painfully slowly) to non-human animals. We do not know if this trend will continue, and we cannot know for sure how it will be affected if we succeed in creating synthetic life. It seems, however, that the most important question is how we treat the life we create, not how it has come about (Douglas et al., 2013; Cabak Redei, 2021).

## Conclusion

If humans acquire the ability to create synthetic life, it will have some very important consequences. It will provide large benefits but also risks. One risk that is sometimes pointed out is the risk that it will diminish the value of life. In this article, we have analyzed five properties that are strongly associated with life, strongly associated with value, and risk being negatively affected when humans learn to create life. The properties that were discussed are originality, origin, mystery, naturalness, and autonomy.

It turned out that even if we accept both that these properties are important grounds for valuing life, and that they will be negatively affected by our ability to create life, then the value of the original life (life 1.0) does not seem to be in danger. On the other hand, it may be that the value of the synthetic life (life 2.0) will be negatively affected, which in turn can lead to a disturbing difference in value between different kinds of life.

On the other hand, we also came to the conclusion that it seems doubtful whether a future human ability to create life will really have a great negative impact on these characteristics. We must also remember that here we have only discussed characteristics that have been selected precisely because they might be negatively affected by a future human ability to create life. There are also many other reasons to value life, reasons that will not be affected at all by a human ability to create life, which means that even if an ability to create life will negatively affect the value of life (or at least affect the value of life 2.0), it will only affect a part of life’s value (including the value of life 2.0).

That said, of course, there are also a number of other concerns regarding synthetic life that need to be looked into before we start creating. How it will affect how we value life is but one of them and it is still true for synthetic life, as it is for all other fields of research: It is not enough to ask, can we do it. We must also ask, should we do it.

## Data Availability Statement

The original contributions presented in the study are included in the article/supplementary material, further inquiries can be directed to the corresponding author.

## Author Contributions

The author confirms being the sole contributor of this work and has approved it for publication.

## Conflict of Interest

The author declares that the research was conducted in the absence of any commercial or financial relationships that could be construed as a potential conflict of interest.
